# Fibro-Melanoid Tumor of the Antrum, Requiring the Extirpation of the Entire Super-Maxillary Bone—Operation—Recovery

**Published:** 1861-02

**Authors:** F. Hinkle

**Affiliations:** Marietta, Pa.


### Selections.

### FIBRO-MELANOID TUMOR OF THE ANTRUM, REQUIRING THE EXTIRPATION OF THE ENTIRE SUPER-MAXILLARY BONE—OPERATION—RECOVERY.

By F. Hinkle, M. D., of Marietta, Pa.

March 9th, 1860. I was called to see Mrs. Wm. G., of Columbia, æt. 35 years, married, the mother of five children—the youngest three years old—and now thinks herself near three months advanced in her sixth pregnancy. She is of healthy parentage, and has always enjoyed excellent health until about two years since, when she was attacked with severe inflammation of the right lachrymal sac and its duct, for which she was treated by different physicians. About a year ago, she noticed a small tumor in the right nostril, which at first increased but slowly; recently, however, it has grown rapidly to its present size. It now fills the entire antrum, the right nostril, from which it protrudes externally, and by displacement of the septum, causes such occlusion of the left, that it is with some difficulty an ordinary probe can be introduced. The nose is much pushed to the left side, and the orbital cavity so encroached upon that the eye is displaced outward and upward, and rests against and upon the supra-orbital ridge. The integuments of the brow are thrown into numerous wrinkles, and upon elevating and retracting the upper lid, the whole globe of the eye seems to stand external to and rest upon the surrounding parts. The pressure upon the nerve has destroyed the sight of the right eye. Recently the pain has much increased in the part and she now suffers great inconvenience from the constant accumulation in the fauces, of a viscid secretion, so tough as to require frequent removal with the fingers to prevent suffocation. The distress from this cause is very great, almost entirely preventing sleep, which gives to the patient a very haggard and worn-out appearance.

Being an intelligent woman, I informed her that nothing short of the entire removal of the disease could afford her relief, and fully explained to her the dangers—now greatly enhanced by her being enciente—of so formidable an operation and also the fact that should she survive the operation, the relief afforded might only be temporary, owing to the possibility of a recurrence of the disease. Satisfied that without relief from her sufferings, a speedy death was certain, she begged that the operation might be performed as early as possible, being "willing to suffer all things that her life might be spared even for a time to her little children." "In my case 'tis die dog or eat the hatchet," was her laconic, but expressive reply, to the artist to whom she was sitting for her picture the day previous to the operation, upon being asked whether she was not afraid of the result of so formidable an operation.

After mature deliberation upon her case, I decided to give her the benefit of an early operation, believing, from the rapid progress the disease had already made, that before the period of gestation would be completed, the poor woman would be beyond the reach of help.

March 22d, noon. Assisted by Drs. Eher, of Lancaster, and McCorkle, of Columbia, the patient being placed under the influence of a mixture of three parts of ether and one of chloroform, I made a semilunar incision from the angle of the mouth to a point midway between the ear and the external canthus of the eye, ligated the facial artery, and proceeded with a rapid dissection of the parts. Just beneath the infra-orbital foramen, I found the tumor protruding through the walls of the antrum, about the size of a dime. This being carefully dissected from the adjacent soft parts, I cleared the infra-orbital ridge and internal canthus, loosing the teguments as far as the tuberosity and median line of the nose, and while the flap was firmly retracted upon the forehead by one of the assistants, I separated the tumor from its attachments within the orbit, as far as they could be reached. The incisor tooth having been extracted two days previous, I now notched the alveolus and hard palate with the saw, and with one blade of the cutting forceps, carefully introduced into the nostril and the other in the notch, divided the bone with a single cut. The malar bone was next freely notched on its under border, and with one blade of the forceps in the notch, and the other in the orbit, was transfixed into the spheno-maxillary fissure, leaving the frontal and orbital processes as a future support to the ball of the eye, and then with one blade of the forceps introduced into the nostril, the division of the bony attachments was completed by cutting through the nasal bones and nasal process of the maxillary into the orbit. Depressing the mass with my left hand, I divided the remaining soft parts with the scalpel, and removed the entire mass of the disease, except a small portion that was closely adherent to the sheath of the nerve at the bottom of the cavity. The parts were next sponged with a strong infusion of matico, which arrested the hemorrhage, after which every remaining particle of diseased structure was carefully removed. After suffering the cavity to remain exposed for some minutes, during which time it was freely sponged with cold water, it was filled with charpie, soaked in the infusion of matico, the flap brought down, and the operation completed by the introduction of five sutures of silver wire, by which the parts were admirably retained in situ. The patient was now removed to her bed, and cold water dressing applied to the part. Being much prostrated, a draught, containing brandy and spr. ammon. aromat. was ordered to be given occasionally, until reaction was established.

5 o'clock, P. M. Reaction fully established; brandy and ammonia discontinued, and brandy panada and milk and egg ordered as nourishment during the night. Patient complains of pain, for which quinia sulph. gr. 2 and morph. sulph. gr. $\frac{1}{4}$, in syr. acaciæ was ordered every three hours until relieved, and a lotion of liq. plumbi. subacetat. dil. and ext. belladonnæ, to be applied over the eye, around which there is some tumefaction and discoloration from effusion of blood. The patient was again seen by Dr. McCorkle, at ten o'clock, and being quite comfortable after taking two doses of the anodyne mixture, it was discontinued.

23d, 9, A. M. Patient has had several hours comfortable sleep during the night, from which she seems quite refreshed. Pulse 90; skin moist. Continue same dressings to the wound, and use beef tea in conjunction with previous diet.

Being unable to see the patient more than once daily, she was taken charge of by Dr. McCorkle, from whom she received every attention between the times of my daily visits.

24th, 10 A. M. Patient is very weak; pulse 100, and feeble; complains of great nausea from the offensive discharge from the wound pouring into the throat; has vomited several times. I removed all the dressing from the cavity, and after washing it well with cold water, filled it again with lint soaked in the infusion of matico, and annointed with caster-oil to prevent its adhering to the part; continued the lotion and cold water dressing externally, and ordered diet of beef tea and milk-punch, and carb. ammon. gr. 2, and morph. sulph. gr. $\frac{1}{8}$, every four hours, in mucilage. At 8 o'clock, P. M., the nausea and occasional vomiting continuing, Dr. McCorkle ordered a saline cathartic.

25th, 10 A. M. Bowels have been moved; vomiting less frequent, but nausea still continues; very weak. Beef tea and brandy to be taken freely, and continue ammonia mixture. At 4 P. M., Dr. McCorkle was summoned to the country, at which time our patient was much more comfortable. Late in the evening, she was induced by a friend to take some warm oyster broth, by which the dressing of the cavity was saturated, and a slight capillary hemorrhage brought on, which gradually increased until about midnight, when, the family becoming alarmed, the doctor was sent for. Finding that the patient was rapidly sinking, and that the readjustment of the dressings, and the introduction of fresh portions of lint, had only temporarily arrested the hemorrhage, I was summoned to his aid, and at 2 o'clock, A. M., of the 26th, found the patient almost in articulo mortis; pulseless, with blood oozing freely from both mouth and nostrils, and frequent retchings, as if to free the stomach of the blood which had found its way into that viscus. I immediately removed from the mouth and fauces large clots of blood, gave a large draught of brandy and ammonia, and as speedily as possible removed all the dressings from the cavity, and by sponging it with the strong infusion of matico, and refilling it with the charpie, saturated with that liquid, succeeded in arresting all bleeding. It is the opinion of Dr. McCorkle, that the patient had lost not far from three pints of blood, and so great was the prostration, that, notwithstanding the active administration of the strongest diffusible and nervous stimulants, her life was despaired of for some hours, during which time she passed from one state of syncope to another, with cold extremities and occasional convulsive paroxysms. There was also constant nausea and severe retching. Ice was given freely, spice plasters applied over the abdomen, and sinapisms and hot alcoholic fomentations to the extremities. 10 o'clock, A. M., nausea and vomiting subsided; patient comfortable, but very weak. Ordered wine and egg, and beef tea and brandy, and left patient in care of Dr. McCorkle.

27th, 8½ o'clock, A. M. Patient has rested pretty well since I last saw her. Complains of some pain in the wound in which there is now considerable tumefaction, and the areolar tissue around the eye is filled with and discolored by the blood effused during the efforts at vomiting. Apply cold poultice of elm bark, ext. arnica and powdered alum over the eye, and continue cold water dressing to the wound.

From this time forward, there was no untoward symptom. The pain, tumefaction, and discoloration, gradually subsided. The patient being very anæmic, I prescribed syr. ferri et quin. cit., with good diet; stimulants as required, gradually substituting malt liquors for the alcoholic stimulants; the bowels kept soluble with mild laxatives when necessary, and the wound regularly dressed. The patient's health and strength gradually improved, and in six weeks from the time of the operation she was able to walk about the house, and, during the seventh week, walked two squares on the street, supporting herself on her daughter's arm.

May 23d, 10 o'clock, P. M. Dr. McCorkle attending, she was delivered of a male child, dead, of about 5½ months. Two days previous she had fever and swelling in her limbs, and from that time felt no further motions of the child. Immediately after her delivery, she said she felt "like a new woman."

May 24th. Pulse 100; skin normal; no fever; tongue clean; appetite good; ate a piece of toast just before my arrival; complexion good; expression cheerful, and general appearance healthy.

From this time she steadily improved in health, and on

July 17th, visited me at my office; she says she now enjoys as good health as she has ever done; is quite cheerful, and were it not for a scar, resulting from the application of some escharotic by an empirical practitioner some months previous to my seeing her, would show but few marks of the severe operation to which she had been subjected.—*Medical and Surgical Reporter.*

